# Immunotargeting of Cervical Cancer Cells with the Adaptor CAR T-Cell Platform RevCAR

**DOI:** 10.3390/ijms27219704

**Published:** 2026-10-29

**Authors:** Nathalia Jones-Cifuentes, Claudia Arndt, Liliana R. Loureiro, Nicole Berndt, Alexandra von Jutrzenka-Trzebiatowski, Anja Feldmann, Jennifer Bachmann, Barbara Seliger, Bladimiro Rincon-Orozco, Michael Bachmann

**Affiliations:** 1 Institute of Radiopharmaceutical Cancer Research, Helmholtz-Zentrum Dresden-Rossendorf (HZDR), 01307 Dresden, Germany; n.jones-cifuentes@hzdr.de (N.J.-C.); l.loureiro@hzdr.de (L.R.L.); n.berndt@hzdr.de (N.B.); a.von-jutrzenka-trzebiatowski@hzdr.de (A.v.J.-T.); a.feldmann@hzdr.de (A.F.); mcbachmann@t-online.de (M.B.); 2 School of Medicine, Department of Basic Sciences, Universidad Industrial de Santander, Bucaramanga 680002, Colombia; 3 Mildred Scheel Early Career Center, Faculty of Medicine Carl Gustav Carus, TUD Dresden University of Technology, 01062 Dresden, Germany; 4 German Cancer Consortium (DKTK), Partner Site Dresden, 01062 Dresden, Germany; 5 National Center for Tumor Diseases (NCT), NCT/UCC Dresden, A Partnership Between DKFZ, Faculty of Medicine and University Hospital Carl Gustav Carus, TUD Dresden University of Technology & Helmholtz-Zentrum Dresden-Rossendorf (HZDR), 01307 Dresden, Germany; 6 Brandenburg Medical School and Faculty of Health Science, Institute for Translational Medicine, 14770 Brandenburg, Germany; bynnej@gmail.com (J.B.); barbara.seliger@uk-halle.de (B.S.); 7 Institute of Pathology, Section Immunopathology, Martin-Luther-University Halle-Wittenberg, 06112 Halle, Germany

**Keywords:** cervical cancer, immunotherapy, prostate stem cell antigen (PSCA), RevCAR T-cells, reverse target modules (RevTMs)

## Abstract

The Prostate Stem Cell Antigen (PSCA) is not only overexpressed in prostate cancer (PC) but also in many other tumors, including cervical cancer (CC). The adaptor chimeric antigen receptor (CAR) platform RevCAR can be used for immunotargeting of PSCA-expressing tumor cells. We found that the CC cell line SiHa natively expresses PSCA. The expression was sufficient for redirection of RevCAR T-cells using recently described RevCAR target modules (RevTMs) directed against PSCA. Like almost all PC cell lines, however, SiHa cells also downregulate their PSCA expression in cell culture. To overcome this limitation, we established artificially PSCA-overexpressing SiHa cells. Retargeting of RevCAR T-cells strictly depends on PSCA-directed RevTMs and PSCA-expressing target cells. It leads to (i) upregulation of CD69, Perforin and Granzyme B, (ii) lysis of target cells with EC_50_ values in the picomolar range, and (iii) cytokine secretion of IFN-γ, TNF-α, GM-CSF and IL-2. Phenotypic analysis shows a prominent increase in memory RevCAR T-cells but only a slight increase in exhausted cells, indicating a low level of tonic signaling of RevCAR T-cells. Together, our data supports an efficient and safe immunotargeting of PSCA-expressing CC cells with the RevCAR technology.

## 1. Introduction

Cervical cancer (CC) is one of the leading causes of cancer-related mortality among women worldwide, accounting for approximately 280,000 deaths in the year 2024 [1]. Persistent infection with high-risk human papillomavirus plays a key role in disease development and is widely recognized as one of the most prevalent sexually transmitted infections [2,3]. Despite advances in screening and prevention, clinical outcomes remain poor for patients with advanced or recurrent disease. Conventional treatments, including surgery, chemotherapy, and radiotherapy, provide only limited benefit [4,5]. Therefore, novel therapy options are urgently needed.

T-cells genetically modified to express chimeric antigen receptors (CAR T-cells) have improved the treatment of hematological malignancies, especially B-cell malignancies, and are now clinically approved, showing durable and antigen-specific responses [6,7]. Until now, however, the application of CAR T-cells against solid tumors has been less successful [8,9]. Several reasons appear to be responsible for the failure of CAR T-cells to target solid tumors, including (i) deploying an artificial CAR gene into T-cells can result in tonic signaling, which can lead to poor anti-tumor efficacy, impaired survival, and reduced persistence [10,11]. Furthermore, it can promote T-cell anergy, exhaustion, and activation-induced cell death (AICD) [10,11,12,13]. In the worst case, constitutive signaling can even turn CAR T-cells into malignant T-cells [14,15,16,17]. (ii) The lack of ideal target antigens for most solid tumors can cause “on-target, off-tumor” effects resulting in destruction of healthy tissues [18,19]. (iii) The immunosuppressive tumor microenvironment of solid tumors is shaped by a plethora of factors, including small molecules (e.g., metabolites such as L-arginine, lactate, extracellular ATP), cytokines, chemokines, microRNAs, interactions between tumor and non-tumor cell types including, for example, macrophages, cancer-associated fibroblasts, or regulatory T-cells (Tregs), checkpoint inhibition, and many more. Together, these factors lead to an inhibition of the entry of immune cells, including CAR T-cells, and to a down-regulation of their function [20,21,22,23,24,25,26,27,28,29,30,31,32,33,34].

To understand the origin of the limitations of CAR T-cells, one must take a closer look at the development of the CAR technology and the common structural components of an artificial CAR gene [35,36,37,38,39,40,41,42,43]. In general, a conventional CAR gene encodes three functional domains, which are connected via spacer units: (i) an extracellular antibody-derived domain, which is required for recognition of the target cell; (ii) a transmembrane domain for transport to the cell surface and anchoring of the artificial receptor into the outer cellular membrane; and (iii) intracellular signaling domains for activation of the CAR T-cell after interaction of the extracellular antibody domain with the target cell and formation of an immune synapse-related structure containing clustered CARs [13,44]. Over the past decades, several CAR designs have been put forward, known as first-, second-, third-, and fourth-generation CARs: (i) First-generation CARs contain only an activation signaling domain. Nowadays, it is, however, generally accepted that T-cells require two signals for full functionality: activation and co-stimulation. Activation in the absence of co-stimulation is even counterproductive and drives T-cells into anergy or even to AICD, resulting in tolerance rather than killing of the target cells. For that reason, (ii) second-generation CARs were introduced, which contain not only an activation domain but also a co-stimulatory domain. (iii) Third-generation CARs contain more than one co-stimulatory domain. (iv) Fourth-generation CARs, also known as TRUCKs, deliver an additional payload, usually a cytokine gene, which helps to strengthen the local immune response.

A common disadvantage of all these conventional CAR T-cells is the lack of functional control after adoptive transfer of the genetically modified immune cells into patients. To overcome this limitation, we and others have developed modular CARs, also known as adaptor CAR platforms [45,46,47,48,49,50,51,52,53,54,55,56,57,58,59,60,61,62,63,64,65]. In contrast to conventional CARs, adaptor CARs recognize a universal epitope, e.g., a peptide sequence or haptens like FITC or biotin, or the whole extracellular domain of another receptor, e.g., the folate receptor. The epitope should not be available on the surface of any healthy cell and should not be immunogenic in humans. For our previously described adaptor CAR platform, UniCAR, we selected two peptide epitope sequences. Both epitope sequences, E5B9 and E7B6, have been taken from the nuclear autoantigen La/SS-B [66,67]. These epitopes were selected because they are cryptic, not accessible on native human La protein, and do not represent B- or T-cell epitopes even in anti-La positive autoimmune patients [67,68]. The epitopes E5B9 and E7B6 are recognized by scFvs constructed from the corresponding anti-La monoclonal antibodies (mAbs) 5B9 or 7B6, respectively [45].

After adoptive transfer into patients, adaptor CAR T-cells are initially inactive. For activation, they require a bispecific linker module, termed either a target module (TM) or adaptor molecule: One arm of this bispecific linker molecule can bind to the adaptor CAR, and the other arm to the cell surface of the tumor cell. Consequently, a cross-linkage between effector and target cell is formed, which finally activates the adaptor CAR and leads to the killing of the target cell. Following this concept, infusion of the TM in a patient turns “on” the adaptor CAR T-cell, while stopping the infusion, followed by the elimination of the TM, will turn “off” the adaptor CAR T-cell. Consequently, the functionality of adaptor CAR T-cells and, in parallel, potential side effects can easily be controlled. Proof of concept for the possibility to switch adaptor CAR T-cells repeatedly “on” and “off” via application of the TM was confirmed in clinical phase one trials for our adaptor CAR systems UniCARs and RevCARs (see below) (NCT04633148, NCT04230265, NCT04633148) [50,51,52].

In summary, adaptor CARs can overcome the lack of regulation of conventional CAR T-cells via a discontinuous application of TMs, which enables the fine-tuning of their function and the management of critical side effects.

However, as most adaptor CAR genes have the same layout as conventional (secondary) CARs, they still harbor the same risk of tonic signaling. In this context, it is important to mention that not only the intracellular signaling portions of CARs but also their extracellular antibody domain can provide a major contribution to tonic signaling [13,67]: Usually, the extracellular antibody domain of a CAR consists of the variable heavy and light chain portions of an antibody which are recombinantly fused to form a single chain fragment variable (scFv). The aspired intramolecular folding of these heavy and light chain fragments restores the antigen-binding domain, the paratope, of the original antibody. However, the light chain of one CAR molecule cannot only bind intramolecularly to the heavy chain domain but can also interact intermolecularly with the heavy chain portion of the neighboring CAR, leading to dimerization and even oligomerization of the CARs in the absence of a target cell. This unwanted extracellular clustering of CAR receptors in the absence of a target antigen enhances the threshold of tonic signaling, thereby reducing the additional signal required for a full activation of the CAR T-cell [13]. As a consequence of this tonic signaling, no mature T- or B-cell will occur in mice experimentally expressing CARs in hematopoietic stem cells [13]. Noteworthy, this scFv-mediated clustering depends on the underlying antibody domains. To reduce or overcome this scFv-mediated risk of tonic signaling, we designed a further adaptor CAR platform, which we termed RevCARs, in which the paratope-recognizing scFv in the CAR gene and the epitope in the TM are replaced in a reversed manner [55].

Therefore, RevCARs differ from all other known adaptor CARs as follows: The extracellular (5B9 or 7B6) scFv domain of UniCARs is substituted by the corresponding small peptide epitopes E5B9 or E7B6, respectively. Vice versa, these peptide epitope sequences, which usually mediate the interaction between UniCAR TMs and UniCARs, are replaced by the corresponding 5B9 or 7B6 scFv domains in RevCAR TMs (RevTMs). Like UniCAR T-cells, RevCAR T-cells can be regulated via application of the RevTM, as already confirmed in a more recent phase 1 clinical trial (NCT04633148) for targeting of CD123 in acute myeloid leukemia (AML) with (E5B9) RevCAR T-cells [50,51,52].

Obviously, TMs of all kinds of adaptor CAR systems not only mediate interaction with the adaptor CAR T-cell, but they also determine the target specificity. Preclinical data support the idea that a sequential or simultaneous application of TMs with different antigen specificity, also known as “OR-gated” targeting, can help to overcome failure of immunotherapy due to loss of antigen. Furthermore, splitting the activation and costimulatory signaling domains into two separate CAR genes enables the recognition of expression patterns, also known as combinatorial or “AND-gated” targeting [46,69,70,71,72]. The small size of RevCAR genes facilitates this concept of “AND-gated” targeting, as two CAR genes can easily be fused in a single vector construct. So, co-transduction of two independent CAR gene constructs is no longer required in the case of Dual RevCARs. Dual RevCAR T-cells will only attack tissues that express two target antigens and spare single-positive cells, as already preclinically shown for combinations of RevTMs, e.g., carcinoembryonic antigen (CEA) with epithelial cell adhesion molecule (EpCAM), epidermal growth factor receptor (EGFR) with the disialoganglioside GD2, prostate stem cell antigen (PSCA) with prostate membrane antigen (PSMA), and CD33 with CD123 [55,73]. Thus, “AND-gated” targeting can further increase tumor specificity and safety of CAR T-cell therapies. Until now, this hypothesis is only supported by preclinical data and still must wait for clinical confirmation until sufficient safety data for mono-targeting has been collected stepwise to convince the legal authorities to allow a clinical application of such a multitargeting study.

In the current study, we aimed to establish the first mono-immunotargeting approach of CC cells using the (E5B9) RevCAR system. Searching the literature for potential immunotargets overexpressed on CC cells and corresponding antibodies suitable for construction of RevTMs, we identified the following TAAs, which are commonly known to be heterogeneously overexpressed on many different solid tumor tissues, including EpCAM [74], Her2 [75], EGFR [76], mesothelin [77,78], sialyl Tn [79], and placental alkaline phosphatase (PLAP) [12,80,81,82]. In addition to these immunotargets, there is accumulating evidence for overexpression of PSCA on CC tissues. PSCA was originally described as a TAA in PC [83,84,85], but since then has also been detected to be expressed on many other solid tumors, including pancreatic cancer and gynecological malignancies, which underlines its evaluation as a potential immunotarget for CC cells [86,87,88,89].

Although we have already developed TM domains against EpCAM, Her2, EGFR, GD2, and sialyl Tn [90], PSCA is of special interest for us as we have previously established an anti-PSCA-directed scFv domain [91] which has already been tested clinically in the format of a bispecific T-cell engaging molecule (NCT03927573), thereby confirming the safety and efficacy of this anti-PSCA domain in tumor patients. Moreover, based on the same anti-PSCA domain, we most recently developed two novel anti-PSCA RevTMs for immunotargeting of PC cells using the RevCAR system [92]. For these reasons, we wanted to evaluate their potential for retargeting PSCA-positive CC cells. While searching for a suitable CC cell culture system, we found that PSCA is expressed by the CC cell line SiHa. Unfortunately, we observed that SiHa downregulates PSCA expression over time in cell culture. A downregulation of PSCA expression was not unexpected, as PSCA expression is also downregulated in almost all cultured PC tumor cell lines (e.g., the commonly used LnCAP cell line [93]). Only a few cell lines (LAPC-9 and RT4) express PSCA in cell culture but in an unreliable manner [94,95]. Therefore, we and others have established reliable PSCA-overexpressing model cell systems for preclinical studies by transduction of PC cells (e.g., PC3 or LnCAP) with the gene encoding PSCA.

In addition to an efficient retargeting of the native PSCA-expressing CC SiHa, we here present (i) the development and characterization of a reliably PSCA-positive CC cell model system by transduction of PSCA in the model CC cell line SiHa, (ii) the retargeting of these PSCA-positive CC model cells with RevCAR T-cells applying the most recently described novel PSCA-directed RevCAR TMs, and (iii) the estimation of potential risks when applying this anti-PSCA RevCAR T-cell system including the analysis of cytokine release and tonic signaling.

## 2. Results

### 2.1. Expression and Characterization of PSCA RevTMs

**Figure 1:**
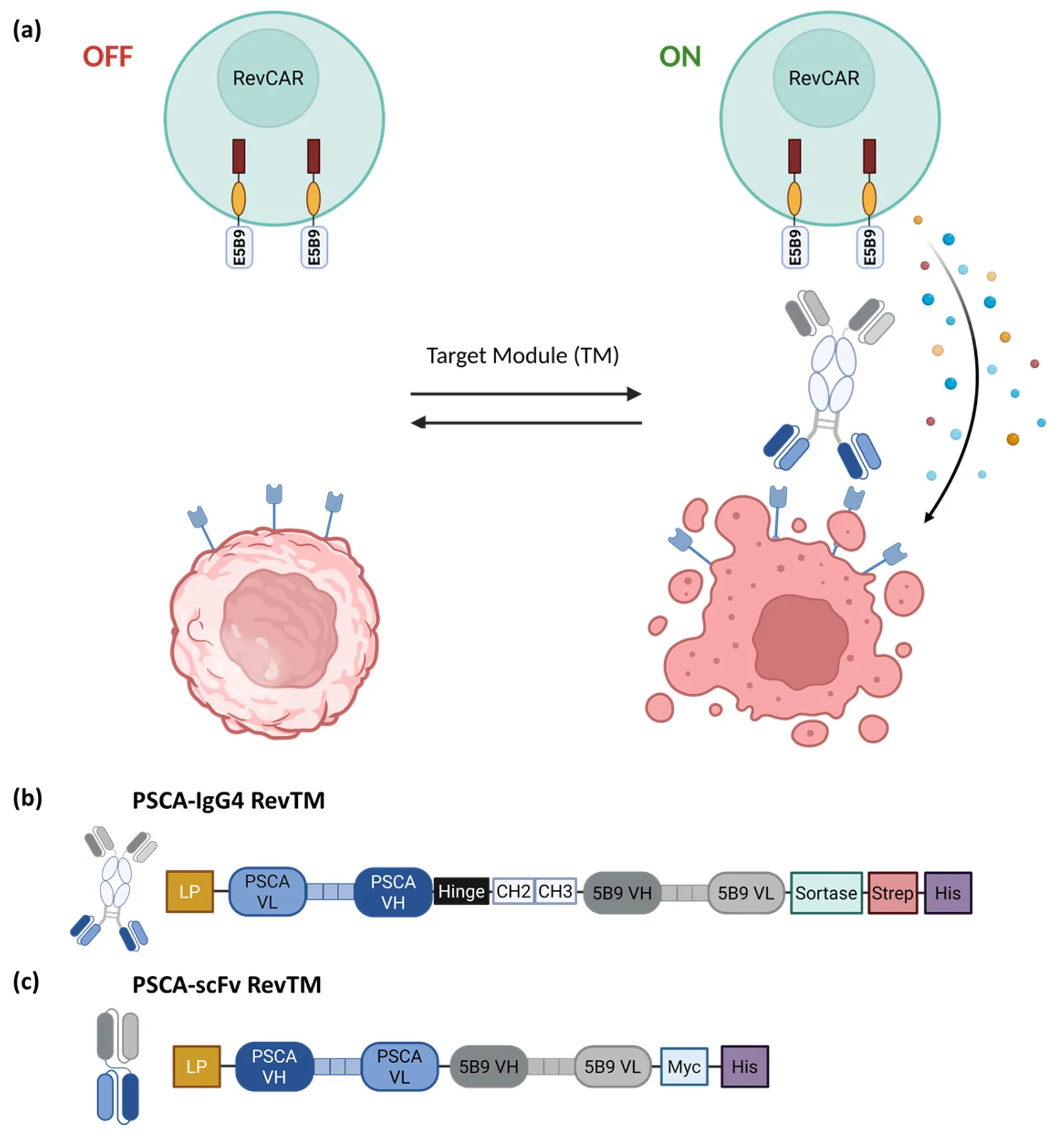
Structure and schematic representation of the RevCAR system and PSCA RevTMs. (**a**) The RevCAR system can be combined with different RevTM formats to specifically redirect RevCAR T-cells toward tumor cells. RevCAR T-cells are activated only in the simultaneous presence of the RevTM and the target cell (ON), whereas in the absence of the RevTM, RevCAR T-cells remain inactive (OFF). RevCAR T-cells express a reverse chimeric antigen receptor (RevCAR) comprising an intracellular CD3ζ signaling domain (red), a CD28 co-stimulatory domain (yellow), a transmembrane and hinge domain (black line), and the peptide epitope E5B9 (light grey) as the extracellular domain. (**b**,**c**) PSCA RevTMs are bispecific molecules with one binding site directed against the E5B9 epitope on RevCAR T-cells and the other directed against the tumor-associated antigen PSCA. Both scFv-based and human IgG4 backbone-based RevTMs can be combined with the RevCAR system. LP, leader peptide; VH, variable domain of the heavy chain; VL, variable domain of the light chain; Sortase, sortase recognition site; Streptactin-tag; His, hexa-histidine tag. Created in BioRender.

As mentioned above, the RevCAR platform consists of two components: (i) genetically modified T-cells expressing a RevCAR and (ii) a bispecific RevTM that mediates target cell recognition and RevCAR T-cell activation. The RevCAR construct is based on second-generation CARs using as the extracellular binding domain the E5B9 epitope derived from the human La protein [45,66], a CD28-derived hinge, transmembrane domain, and intracellular CD28 and CD3ζ signaling domains as schematically summarized in Figure 1a. In this system, target cell recognition and subsequent T-cell activation depend on RevTM-mediated crosslinking between RevCAR T-cells and tumor cells. In principle, all RevTMs are bispecific antibodies recognizing, on the one hand, the peptide epitope E5B9 and, on the other hand, a TAA, in this case PSCA. For this study, two recently developed PSCA-specific RevTMs referred to as PSCA RevTMs were used [45,66]. These differ in their structural formats, one being an scFv-based RevTM, while the other is built based on an IgG4 backbone (Figure 1b,c). The IgG4 backbone contains three-point mutations to minimize Fcγ receptor (FcγR) binding and to reduce unintended interactions with FcγR-expressing cells [96]. All constructs contain an N-terminal Igκ leader sequence to promote RevTM secretion and C-terminal tag sequences for purification and detection.

Both PSCA RevTMs contain a PSCA-specific binding domain composed of humanized VH and VL domains derived from the murine anti-PSCA mAb MB1 [91], linked by flexible glycine–serine linkers. The IgG4-based format provides a larger molecular scaffold, whereas the scFv-based RevTM represents a smaller format. In the IgG4-based construct, the anti-PSCA scFv was fused to the hinge and Fc region of human IgG4 and combined with La-specific VH and VL domains of the murine mAb 5B9 to enable RevCAR engagement (Figure 1b,c).

Stable producer 3T3 cell lines were used to express PSCA RevTMs. Both PSCA RevTMs were purified from culture supernatants by affinity chromatography. SDS-PAGE under reducing conditions revealed a predominant band at approximately 85 kDa for the PSCA-IgG4 RevTM and approximately 55 kDa for the PSCA-scFv RevTM, consistent with their expected monomeric sizes (Supplementary Figure S1a). Immunoblotting using an anti-His antibody confirmed protein identity (Supplementary Figure S1b). Based on the IgG4 hinge region, PSCA-IgG4 RevTMs are expected to form disulfide-linked dimers under native conditions, corresponding to a theoretical molecular weight of 166 kDa. These results confirm the successful production of both PSCA RevTM formats. In agreement with the analysis of previously described TMs and RevTMs, the purity of the isolated RevTMs is sufficient for subsequent preclinical functional studies.

### 2.2. Bispecific Binding of Both PSCA RevTMs to RevCARs T-Cells and PSCA-Positive CC Cells

PSCA was initially identified as a potential tumor target in PC tissues and was later found to be expressed in many other tumor tissues, including in CC [86,87]. Interestingly, we observed a native expression of PSCA in the parental CC cell line SiHa. The native expression of PSCA in the CC cell line SiHa was not influenced by transduction of the cells with the reporter gene encoding firefly luciferase. However, the native PSCA expression in both the luciferase-modified (SiHa-Luc^+^) and the unmodified parental SiHa cells was not reliable and dramatically declined over time. The decrease in PSCA expression over time estimated for SiHa-Luc^+^ cells is shown in Supplementary Figure S2. The proportion of PSCA-positive SiHa-Luc^+^ cells decreased progressively from 58% after 1 week to 30% after 2.5 weeks and 3% after 8 weeks in culture (Supplementary Figure S2).

Downregulation of the native expression of PSCA in cultured cell lines was not unexpected: Similarly, PSCA expression is downregulated in almost all commonly used PC cell lines (see Introduction section). In previous studies of the immunotherapy of PC cells targeting PSCA, we and others have therefore used cell lines transduced to overexpress PSCA. Following the same strategy, we established a PSCA-overexpressing SiHa-Luc^+^ cell line, termed the SiHa-PSCA^+^-Luc^+^ cell line.

**Figure 2:**
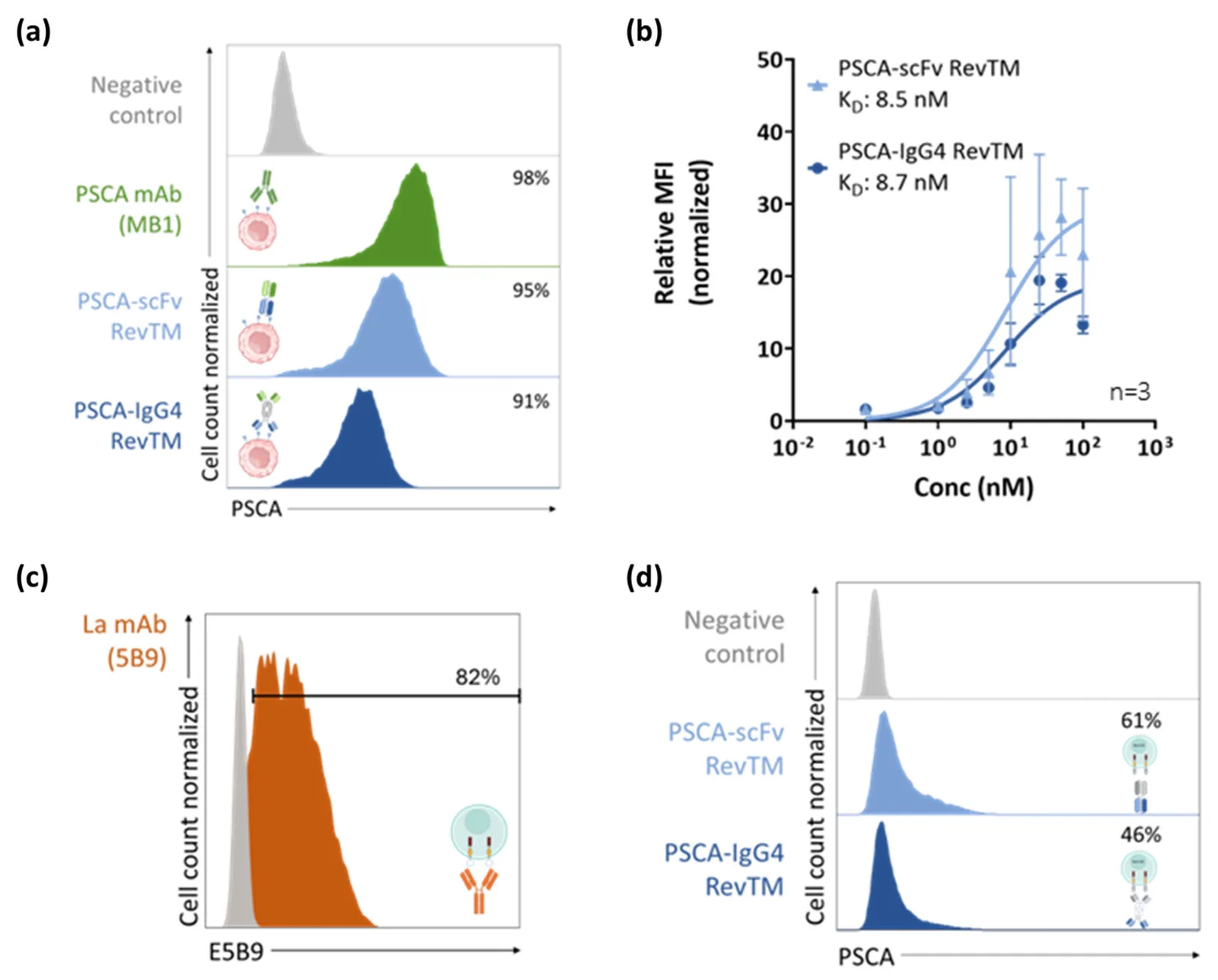
Bispecific binding of PSCA RevTMs to SiHa-PSCA^+^-Luc^+^ cells and RevCAR T-cells. (**a**) PSCA expression on SiHa-PSCA^+^-Luc^+^ cells was assessed by flow cytometry using the anti-PSCA mAb MB1 or PSCA-scFv- or PSCA-IgG4 RevTMs. Binding was detected using Alexa Fluor 647 goat anti-mouse IgG antibodies or APC-conjugated anti-His mAbs, respectively. An isotype-matched control antibody served as the negative control. (**b**) In addition, target cells were incubated with decreasing concentrations of PSCA RevTMs, and binding was detected using an anti-His antibody to generate titration curves for K_D_ calculation. Experiments were performed in technical triplicate and independently repeated at least three times. Data are presented as relative median fluorescence intensity (MFI) ± SD. (**c**) Expression of the extracellular RevCAR receptor domain (E5B9) on transduced T-cells was confirmed using the anti-La mAb 5B9 followed by Alexa Fluor 647 goat anti-mouse IgG. Cells incubated with the secondary antibody only served as a negative control. (**d**) Binding of the bispecific PSCA RevTMs to the extracellular E5B9 domain on RevCAR T-cells was detected using an APC-conjugated anti-His mAb. An isotype-matched control antibody served as the negative control. Data are displayed as histograms, and binding results are expressed as the percentage of positively stained cells under the His marker.

Using SiHa-PSCA^+^-Luc^+^ and RevCAR T-cells, we estimated the binding capability of the two PSCA RevTMs by flow cytometry. Both the scFv-based and the IgG4-based PSCA RevTMs bound to SiHa-PSCA^+^-Luc^+^ cells with binding comparable to the parental murine anti-PSCA mAb MB1, which was used as a positive control (Figure 2a). Quantitative analysis revealed high proportions of PSCA-positive cells stained by the PSCA RevTMs, with binding observed in 91% of SiHa-PSCA^+^-Luc^+^ cells for the PSCA-IgG4 RevTM and 95% for the PSCA-scFv RevTM (Figure 2a).

Binding affinity toward PSCA was further validated by titration experiments on SiHa-PSCA^+^-Luc^+^ cells, generating saturation binding curves. Equilibrium dissociation constants (K_D_) of approximately 8–9 nM were obtained for both PSCA-IgG4 and PSCA-scFv RevTMs (Figure 2b). In addition, the interaction of the PSCA RevTMs with the E5B9 epitope on RevCAR T-cells was determined. Both PSCA RevTMs recognized RevCAR T-cells (Figure 2c). The specific binding to the RevCAR epitope E5B9 on RevCAR T-cells was verified using the anti-La mAb 5B9 as a reference control. Overall, 82% of the cells were stained positive for the 5B9 mAb control, while 61% and 46% were found positive for the PSCA-scFv and PSCA-IgG4 RevTMs, respectively (Figure 2c). Both bispecific PSCA RevTMs recognize their respective binding partner on PSCA-expressing target cells and RevCAR-expressing effector T-cells. Collectively, these results confirm specific and high-affinity binding to PSCA-positive targets as well as to RevCAR T-cells.

### 2.3. In Vitro Cytotoxicity of RevCAR T-Cells Redirected Toward SiHa-PSCA^+^-Luc^+^ Cells

To evaluate the capability of the PSCA RevTMs to redirect RevCAR T-cells toward PSCA-expressing CC cells, RevCAR T-cells were co-cultured with SiHa-PSCA^+^-Luc^+^ target cells in the presence or absence of the PSCA RevTMs. Tumor cell killing was quantified using a luminescence-based cytotoxicity assay.

**Figure 3:**
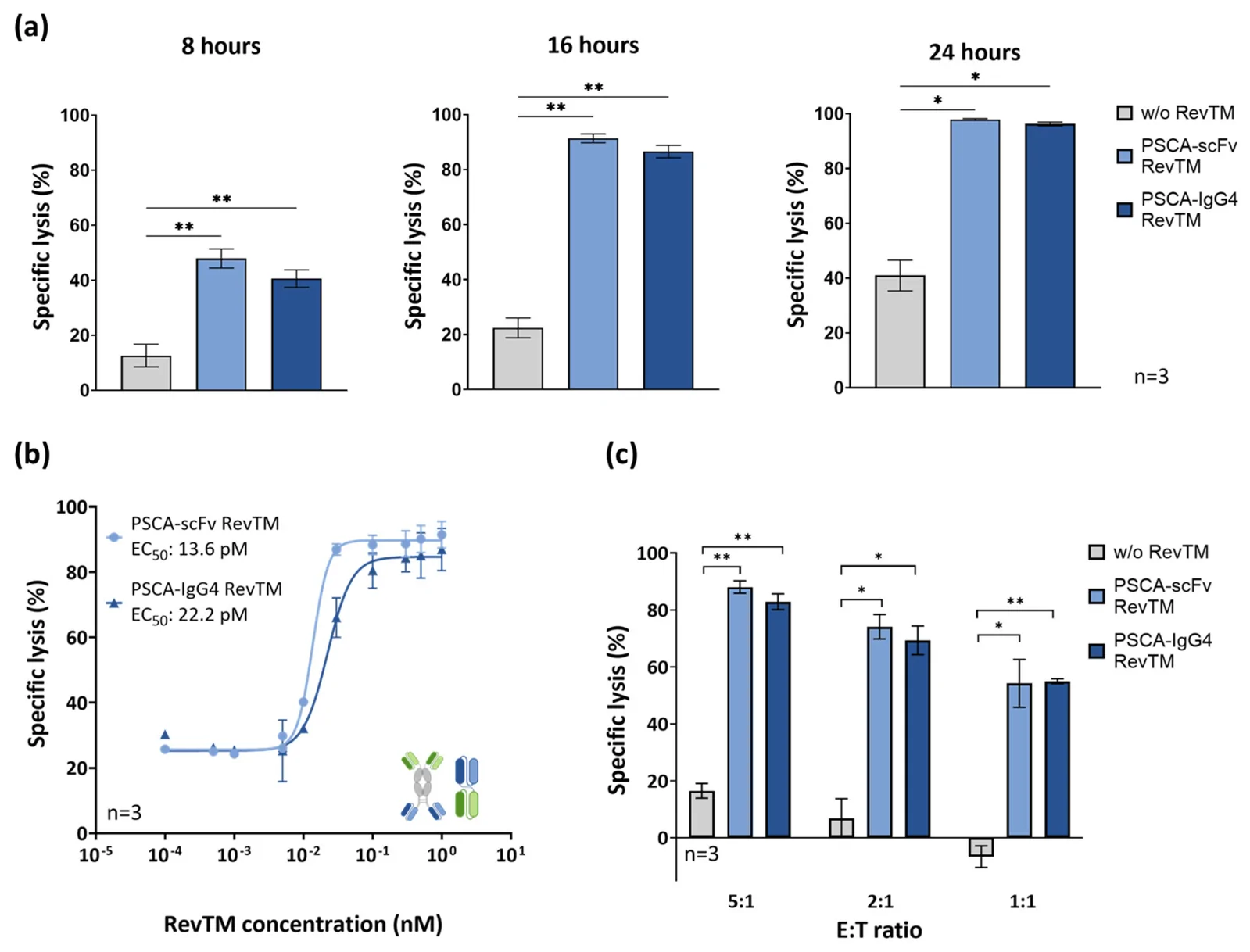
Cytotoxic activity of RevCAR T-cells redirected against SiHa-PSCA^+^-Luc^+^ cells. SiHa-PSCA^+^-Luc^+^ cells were co-cultured with RevCAR T-cells in the absence or presence of PSCA RevTMs. Specific lysis was measured using a luciferase-based cytotoxicity assay. (**a**) Time-dependent cytotoxicity was assessed after 8, 16, and 24 h of co-culture. (**b**) Target cells were co-cultured with RevCAR T-cells (E:T = 5:1) in the presence of increasing concentrations of PSCA RevTMs. EC_50_ values were determined by nonlinear regression analysis using GraphPad Prism version 10. (**c**) Target cells and RevCAR T-cells were co-cultured at different E:T ratios in the presence or absence of PSCA RevTMs. EC50 and E:T ratio assays were performed following a 16 h incubation period. All experiments were performed in technical triplicates and independently repeated using RevCAR T-cells derived from three different healthy donors (n = 3). Data are represented as mean ± SD, and statistical significance was assessed using paired, two-tailed Student’s *t*-tests (* *p* < 0.05, ** *p* < 0.01 compared with the condition without PSCA RevTM).

To assess the kinetics of RevCAR T-cell-mediated cytotoxicity, lysis of SiHa-PSCA^+^-Luc^+^ cells was evaluated after 8, 16, and 24 h of co-culture (Figure 3a). Rapid cytotoxic activity was observed, with approximately 50% lysis detected after 8 h and more than 90% tumor cell elimination after 16 h under the tested conditions. Extending the incubation time to 24 h resulted in only minor additional lysis. Comparable killing kinetics were observed for both PSCA RevTMs, with no significant differences between formats. Based on these findings, a 16 h co-culture period was used for subsequent experiments, with cytotoxic activity remaining dependent on the presence of PSCA RevTMs and minimal background observed in their absence.

In order to rule out that the artificial overexpression of PSCA has an influence on the lysis assay, targeting experiments were also performed using the parental SiHa-Luc^+^ cells, which were cultured for one week. At this time point, about 58% of the cell population expressed PSCA (see also Supplementary Figure S2). As shown in Supplementary Figure S3, both PSCA RevTMs efficiently redirected RevCAR T-cells also to the native PSCA-expressing CC cells.

To further assess the antigen specificity of RevCAR T-cell-mediated cytotoxicity, additional assays were performed using another previously described RevTM that is directed against PD-L1. PD-L1 expression was not detected on SiHa-PSCA^+^-Luc^+^ cells under the experimental conditions. After 16 h of co-culture, the PD-L1-specific RevTM induced a low level of target cell lysis comparable to that observed in the absence of a RevTM (n = 3; Supplementary Figure S4). These findings support the antigen-dependent activity of the PSCA RevTMs and indicate that the presence of a RevTM alone was insufficient to induce substantial target cell killing.

Dose–response analyses using increasing concentrations of PSCA RevTMs confirmed efficient redirection of RevCAR T-cells, with half-maximal effective concentration (EC_50_) values in the low picomolar range (13.6 pM for the PSCA-scFv RevTM and 22.2 pM for the PSCA-IgG4 RevTM) (Figure 3b). In addition, the cytotoxic activity of RevCAR T-cells was evaluated at different effector-to-target cell (E:T) ratios (Figure 3c), demonstrating efficient and statistically significant tumor cell lysis even at low E:T ratios, including 1:1.

### 2.4. Pro-Inflammatory Cytokine Secretion by PSCA-Redirected RevCAR T-Cells

Beyond tumor cell killing, the secretion of the pro-inflammatory cytokines GM-CSF, TNF-α, IL-2 and IFN-γ was examined, as these mediators play relevant roles in both antitumor activity and immune activation but also may cause deleterious side effects known as CRS. For this purpose, RevCAR T-cells were co-cultured with SiHa-PSCA^+^-Luc^+^ cells in the presence or absence of the corresponding RevTMs, and cell-free supernatants were collected after 16 h of co-culture. Cytokine concentrations were subsequently quantified by ELISA.

**Figure 4:**
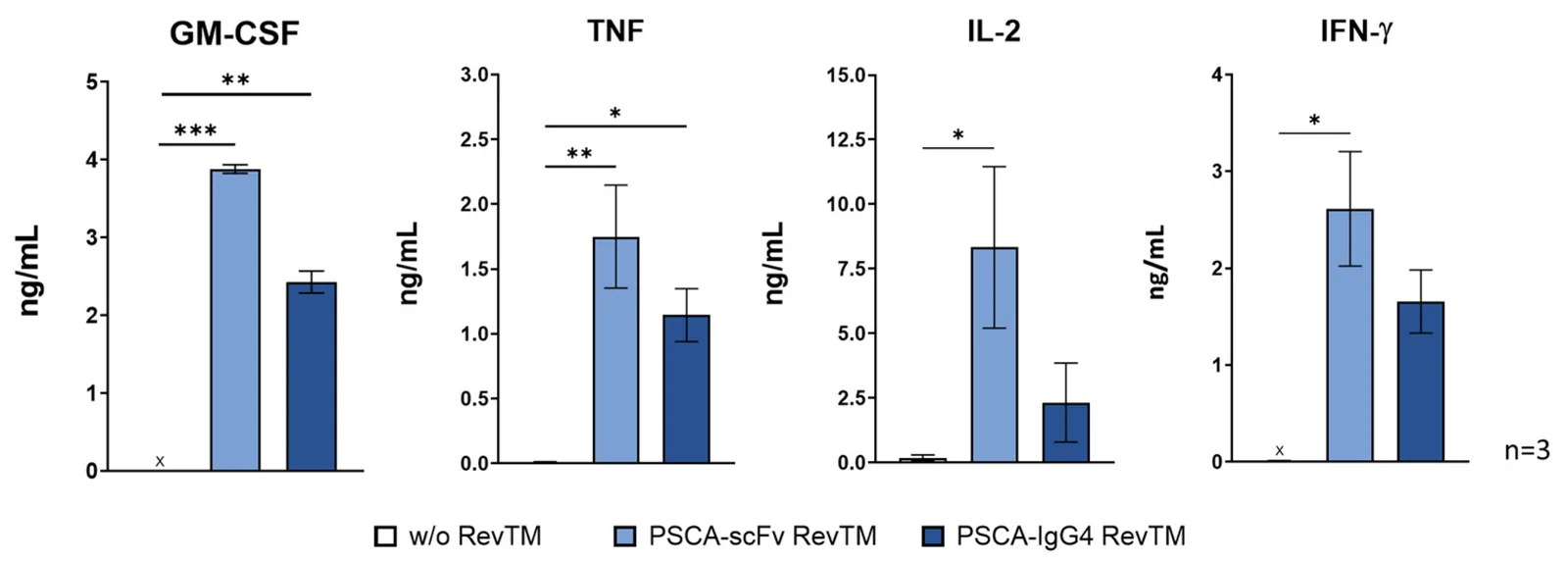
Pro-inflammatory cytokine secretion by RevCAR T-cells redirected via PSCA RevTMs. RevCAR T-cells were co-cultured with SiHa-PSCA^+^-Luc^+^ cells at an E:T ratio of 5:1 in the absence or presence of 50 nM PSCA RevTMs for 16 h. Supernatants from three independent experiments performed using RevCAR T-cells derived from three different healthy donors (n = 3) were collected and analyzed by ELISA for GM-CSF, TNF, IL-2, and IFN-γ secretion. Data are presented as mean cytokine concentration (ng/mL) ± SD. Statistical significance was assessed using paired two-tailed Student’s *t*-test (* *p* < 0.05, ** *p* < 0.01, *** *p* < 0.001). X indicates values below the limit of detection.

As shown in Figure 4, RevCAR T-cells released these cytokines upon PSCA-dependent redirection via PSCA RevTMs, whereas cytokine levels remained low or undetectable in the absence of PSCA RevTMs.

### 2.5. Antigen-Dependent Activation and Acquisition of a Cytotoxic Effector Phenotype by RevCAR T-Cells

As summarized in the Introduction, the impetus for the development of RevCAR T-cells was to reduce the risk of tonic signaling caused by clustering of neighboring CARs on the surface of scFv-based CAR T-cells. In order to further characterize the functional status of RevCAR T-cells following antigen-specific stimulation, the expression of the early activation marker CD69 together with the intracellular cytotoxic effector molecules Granzyme B and Perforin was analyzed by flow cytometry after 16 h of co-culture with SiHa-PSCA^+^-Luc^+^ cells in the presence or absence of the corresponding PSCA RevTMs.

**Figure 5:**
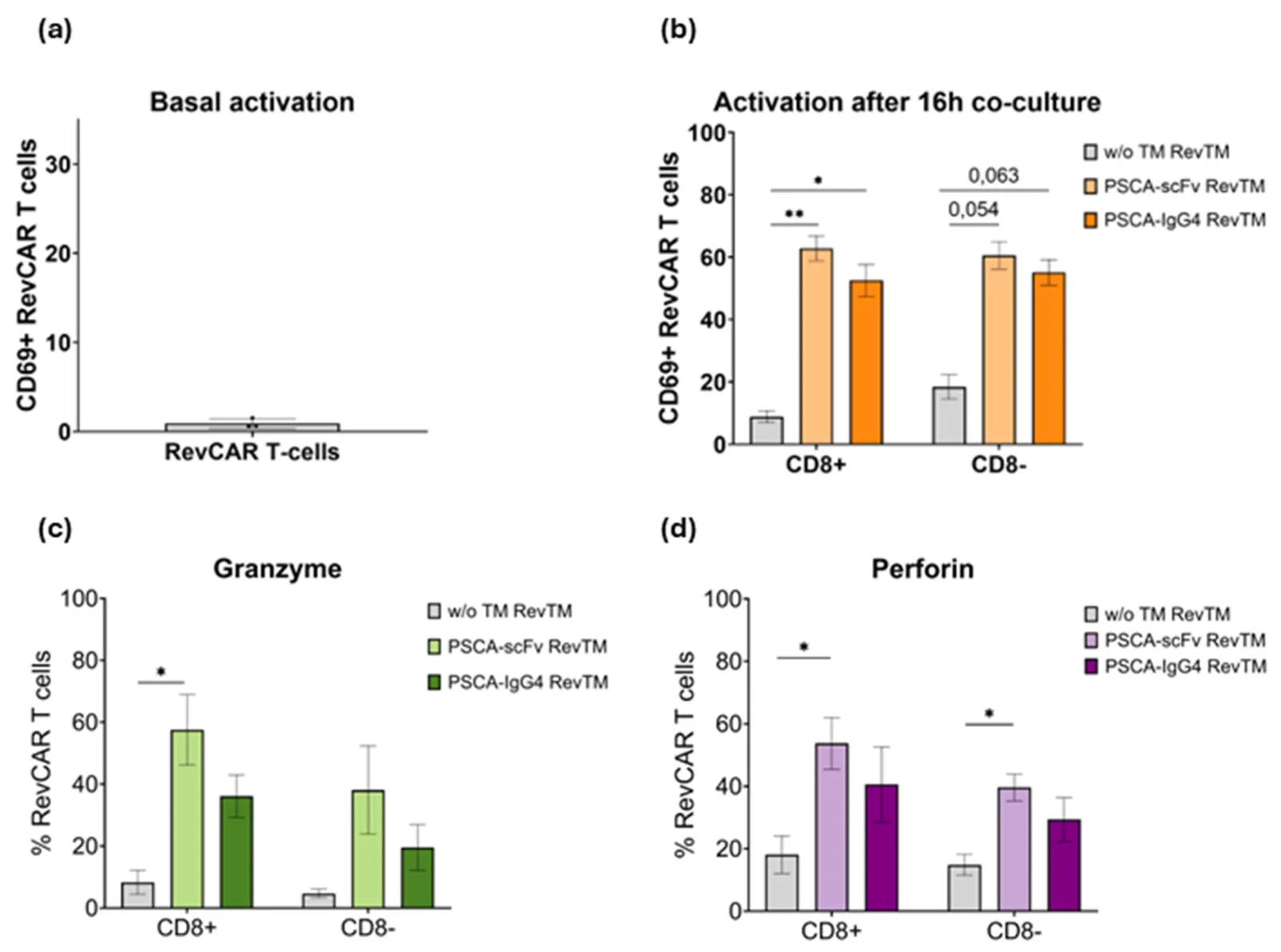
Antigen-dependent activation and acquisition of a cytotoxic effector phenotype by RevCAR T-cells. CD69 expression before (**a**) and after (**b**) 16 h of co-culture with SiHa-PSCA^+^-Luc^+^ cells in the presence or absence of the corresponding PSCA RevTMs. Intracellular Granzyme B (**c**) and Perforin (**d**) expression after 16 h of co-culture under the same conditions. Values represent the mean ± SEM of three independent donors (n = 3). Statistical significance was determined using one-way repeated-measures ANOVA followed by Dunnett’s multiple comparisons test (* *p* < 0.05, ** *p* < 0.01).

Before co-culture, RevCAR T-cells exhibited low basal CD69 expression. After 16 h of antigen-specific stimulation, both PSCA RevTMs significantly increased the frequency of CD69+ cells within the CD8+ RevCAR T-cell population compared with cultures lacking RevTM (Figure 5a,b; scFv, *p* = 0.0099; IgG4, *p* = 0.0190). In contrast, only a trend toward increased CD69 expression was observed in the CD8-population.

Antigen-dependent activation was accompanied by increased intracellular expression of the cytotoxic effector molecules Granzyme B and Perforin. Within the CD8+ RevCAR T-cell subset, stimulation with the PSCA-scFv RevTM significantly increased the frequency of Granzyme B-positive (*p* = 0.0334) and Perforin-positive (*p* = 0.0445) cells compared with cultures lacking RevTM. A comparable response was observed following stimulation with the PSCA-IgG4 RevTM; however, these differences did not reach statistical significance.

Within the CD8-population, stimulation with the PSCA-scFv RevTM likewise resulted in a significant increase in Perforin expression (*p* = 0.0372), whereas Granzyme B expression showed only a non-significant trend toward higher levels (Figure 5c,d).

### 2.6. Phenotypic Characterization of RevCAR T-Cells After Antigen-Specific Stimulation

To further characterize the differentiation status of RevCAR T-cells after antigen-specific stimulation, memory subsets and the expression of exhaustion-associated markers were analyzed following 16 h of co-culture with SiHa-PSCA^+^-Luc^+^ target T-cells in the presence or absence of the corresponding PSCA RevTMs.

**Figure 6:**
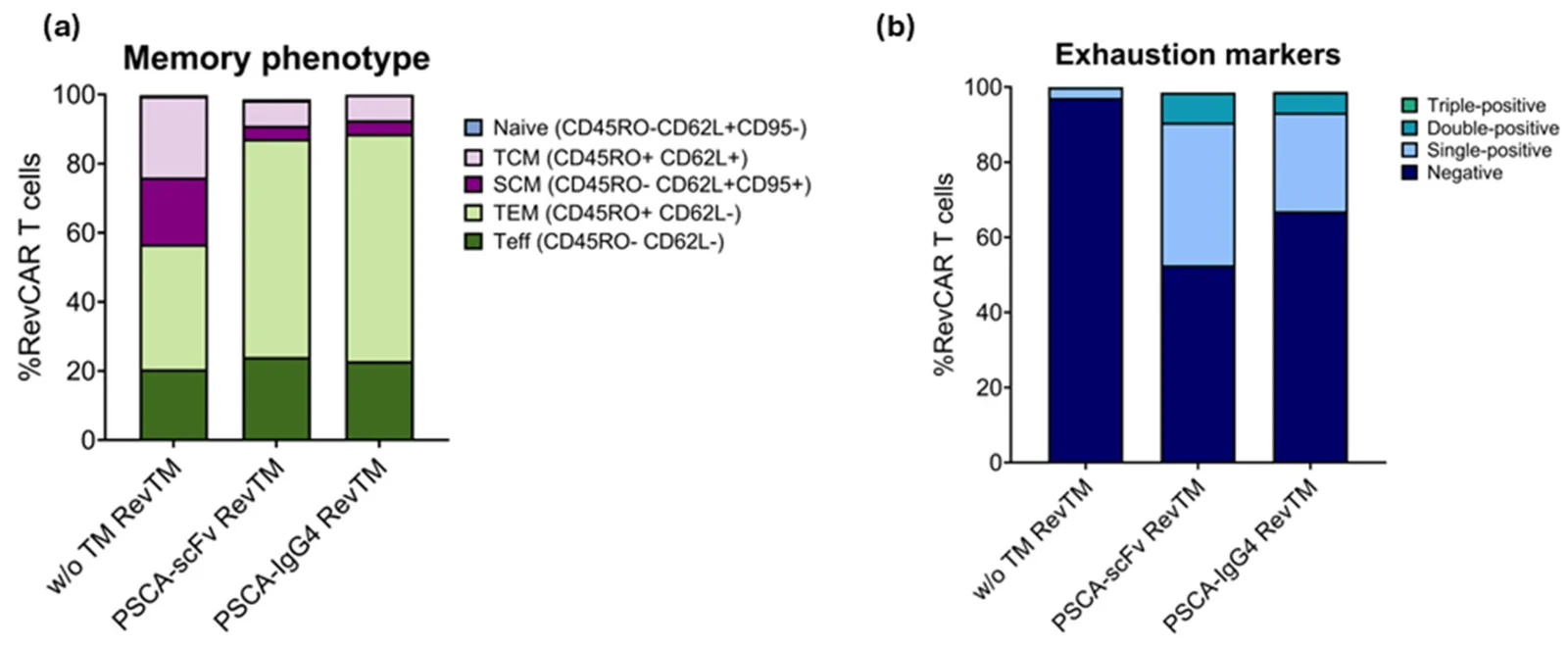
Antigen-induced changes in memory phenotype and exhaustion-associated marker expression of RevCAR T-cells. (**a**) Distribution of naïve (TN, blue), stem cell memory (TSCM, purple), central memory (TCM, light pink), effector memory (TEM, light green), and effector (TEFF, dark green) RevCAR T-cell subsets after 16 h of co-culture with SiHa-PSCA^+^-Luc^+^ cells in the presence or absence of the corresponding PSCA RevTMs. (**b**) Distribution of RevCAR T-cells expressing none (negative, dark blue), one (single-positive, light blue), two (double-positive, turquoise), or three (triple-positive, green) exhaustion-associated markers (PD-1, TIM-3, and LAG-3) under the same experimental conditions. The naïve (blue) and triple-positive (green) populations were assessed but are not visible in the respective graphs because their frequencies were too low. Data represents the mean percentage of three independent donors (n = 3).

RevTM-mediated stimulation significantly increased the frequency of effector memory (TEM) RevCAR T-cells (Figure 6a). This effect was observed with both the PSCA-scFv RevTM (**, *p* < 0.0001) and the PSCA-IgG4 RevTM (*, *p* = 0.0008), indicating a shift in the differentiation profile toward an effector memory phenotype. No relevant changes were detected in the remaining memory subsets.

The expression of the exhaustion-associated markers PD-1, TIM-3, and LAG-3 was subsequently assessed by combinatorial analysis. Most RevCAR T-cells either lacked expression of exhaustion-associated markers or expressed only a single marker after 16 h of antigen-specific stimulation (Figure 6b). Although stimulation with the PSCA-scFv RevTM significantly reduced the proportion of cells lacking exhaustion-associated marker expression compared with unstimulated conditions (**, *p* = 0.0006), only a small fraction of RevCAR T-cells co-expressed two or three markers. A similar distribution was observed after stimulation with the PSCA-IgG4 RevTM, although the differences were not statistically significant.

Taken together with the increased expression of CD69, Granzyme B, and Perforin described above, these results show that RevTM-mediated antigen recognition is associated with the acquisition of a functional cytotoxic effector phenotype characterized by early T-cell activation, enhanced cytotoxic potential, and differentiation toward an effector memory phenotype. The low expression of exhaustion-associated markers also supports the predicted and previously confirmed low risk of tonic signaling of RevCAR T-cells.

## 3. Discussion

As current treatment options for advanced and recurrent CC are highly limited, immunotherapeutic strategies based on mAbs or antibody derivatives [74,97,98,99,100], antibody-drug conjugates (ADCs) [101,102], or adoptive cell therapies using conventional CAR T-cells [12,103,104,105,106] are being actively explored.

CAR T-cell therapies are highly efficient for the treatment of B-cell leukemias. Preclinical studies also show promising results for the treatment of solid tumors; however, the clinical efficacy of CAR T-cell treatments of solid tumors is still inefficient. Several factors may be responsible for the poor outcome of CAR T-cell treatment of solid tumors, including antigen heterogeneity, lack of ideal immunotargets leading to “on-target, off-tumor” effects, limited T-cell infiltration into tumor tissues and killing efficacy due to the immunosuppressive TME, and loss of CAR T-cells due to exhaustion caused by permanent activation through tonic signaling [10,11,12,13,107].

Adaptor CAR T-cell approaches may help to overcome some of these challenges by enabling externally controllable and adaptable T-cell activation via application of a TM. The more recently described RevCAR platform may have additional advantages: Due to the lack of an extracellular scFv domain, RevCARs are not able to cluster on the T-cell surface via the interaction of variable heavy and light chain portions of neighboring CARs and should thereby have a reduced risk of tonic signaling. Like all adaptor CAR T-cell platforms, RevCARs can be used for targeting of multiple antigens either in parallel or sequentially (also known as “OR-gated” targeting). A special advantage of the RevCAR system is the small size of a RevCAR gene, which has already allowed us to construct dual RevCAR gene vectors that encode two different RevCARs, one responsible for co-stimulation and the other for activation, and their use for “(N)AND- and OR-gated” targeting strategies [73]. Such gated targeting strategies may allow in the future to establish multispecific immunotargeting applications to overcome antigen heterogeneity and the immunosuppressive TME [108,109,110]. For a first proof of concept, here, we wanted to establish a monospecific immunotargeting strategy for redirecting RevCAR T-cells to CC cells.

Potential immunotargets of CC include HPV virus-related epitopes [111,112,113] or more commonly expressed TAAs, including, for example, Her2, EGFR, mesothelin, EpCAM, PALP, and PSCA, as summarized in the introduction. PSCA has originally been described as a TAA overexpressed in PC [83,114,115]. Extensive follow-up studies showed an overexpression of PSCA in many other tumor entities, including bladder carcinoma, pancreatic tumors, breast cancer, non-small cell lung cancer, and colorectal cancer [86,94,116,117,118,119,120]. In addition, PSCA has been described as a potential immunotarget in CC [88]. Publicly available TCGA-CESC datasets analyzed through UALCAN, as well as data from the Human Protein Atlas, also support PSCA expression in CC samples, with significantly higher expression levels compared to normal cervical tissues. Given the difficulties associated with culturing primary human CC cells, most studies rely on established cell line models [121,122]. Interestingly, we found that the CC line SiHa expresses PSCA. Unfortunately, this native PSCA expression in cultured SiHa cells is not stable and is rapidly downregulated. Nonetheless, the native expression of PSCA was sufficient for retargeting of RevCAR T cells using the most recently described novel PSCA-directed RevTMs [92]. These PSCA-directed RevTMs are based on the previously described anti-PSCA antibody MB1 [91]. For its anti-PSCA domain, specificity, safety, and efficacy have already been confirmed in a clinical phase 1 trial (NCT03927573).

Obviously, the downregulation of PSCA expression limits the use of the native SiHa cells as a reliable CC cell line model. Downregulation of PSCA expression in cultured tumor cells is not unexpected but well known, including for most PC cell lines. Additionally, regarding PSCA expression in cultured SiHa cells, we are aware of only a few other cell lines that natively express PSCA in cell culture (LAPC-9, RT4) [94,95]. To establish reliable cell culture systems to collect the preclinical data requested by the legal authorities, including for the above-mentioned clinical phase 1 trial for immunotargeting of PSCA-expressing prostate cancer cells (NCT03927573), we previously established PC cell lines that artificially but reliably overexpress PSCA. Following this strategy, accepted by the legal authorities, we here describe the development of the first CC cell culture model for retargeting RevCAR T-cells to PSCA-expressing CC cells by transducing SiHa cells to overexpress PSCA.

Until recently, RevTMs targeting PSCA have predominantly been based on scFv formats, yielding relatively small molecules with favorable tissue penetration and rapid systemic clearance [55,94]. Their short serum half-life enables precise temporal control of adaptor CAR activity, which may be particularly advantageous in settings requiring rapid modulation of T-cell function. More recently, alternative TM formats incorporating Fc domains, such as IgG4-based constructs, have been explored [123], including in the context of PSCA-targeting approaches [92]. These larger formats provide increased molecular stability and prolonged systemic exposure, while maintaining the switchable properties of the adaptor CAR system [90,96]. Mutations in the Fc region of these molecules were introduced to improve their stability and minimize unwanted interactions with Fcγ receptors, thereby reducing the risk of “off-target” immune activation [96,124].

The data presented here show that both novel PSCA RevTM formats have a high binding affinity to SiHa-PSCA^+^-Luc^+^ cells as well as to RevCAR T-cells, with K_D_ values in the low nanomolar range, confirming strong and specific interaction with both molecules.

Cytotoxic kinetics showed rapid tumor cell lysis, with maximal effects achieved after 16 h of co-culture, reflecting efficient activation of RevCAR T-cells in this model.

Furthermore, dose–response analyses revealed that comparable concentrations of PSCA-scFv and PSCA-IgG4 RevTMs were able to induce efficient and specific tumor cell lysis, with EC_50_ values in the picomolar range, indicating strong functional engagement between RevCAR T-cells and their targets. In addition, RevCAR T-cells maintained cytotoxic activity even at low E:T ratios, including 1:1, which is particularly relevant in the context of solid tumors, where T-cell infiltration is often limited and suboptimal [108]. In CC, the tumor microenvironment (TME) imposes both physical and immunological barriers that restrict efficient effector cell access [109,125], resulting in unfavorable local E:T ratios [126,127]. In this setting, the observed cytotoxic activity suggests that PSCA RevTM-mediated cross-linking can still promote effective T-cell-tumor interactions even under conditions that more closely resemble the in vivo TME.

Consistent with these findings, RevCAR T-cells released pro-inflammatory cytokines, including IFN-γ, TNF-α, GM-CSF, and IL-2, upon PSCA-dependent redirection, whereas cytokine secretion remained at baseline levels in the absence of PSCA RevTM, supporting the switch-dependent nature of the system [25]. In addition to cytokine secretion, RevCAR T-cell activation was further evidenced by the induction of CD69, which was accompanied by increased intracellular Granzyme B and Perforin expression, consistent with the acquisition of a cytotoxic effector phenotype. These observations are in line with previous studies using RevCAR platforms targeting, e.g., CD19, ADAMTS13, and PD-L1, in which RevTM-mediated cross-linking similarly induced antigen-dependent secretion of pro-inflammatory cytokines together with CD69 upregulation and increased expression of cytotoxic effector molecules, highlighting the strict target- and RevTM-dependent activation of the RevCAR system [128].

In addition to promoting immediate effector functions, antigen-specific stimulation also induced phenotypic changes, most notably an increased proportion of effector memory (TEM) cells, indicating differentiation toward an activated effector state. Similar differentiation has been observed in PD-L1-directed RevCAR T-cells following antigen-specific stimulation, suggesting that productive RevTM-mediated signaling promotes the acquisition of an effector phenotype [128].

In parallel to this phenotypic shift, RevCAR T-cells exhibited only limited expression of the exhaustion-associated markers PD-1, TIM-3, and LAG-3 after 16 h of co-culture. Together, our phenotypic analysis suggests that acute RevTM-mediated stimulation induced functional activation without evidence of pronounced acquisition of an exhaustion-associated phenotype. This interpretation is consistent with studies demonstrating that expression of individual inhibitory receptors may accompany physiological T-cell activation, whereas functional exhaustion is associated with sustained co-expression of multiple inhibitory receptors [129,130]. Our phenotypic analysis data are also in line with data reported for immunotargeting of CC using conventional CAR T-cells directed against PLAP [12]. In their study, Yekehfallah et al. associated this phenotypic pattern as an indication of low tonic signaling of their CAR construct. Following their interpretation, the presented phenotypic data of PSCA-redirected RevCAR T-cells also support our hypothesis that the RevCAR gene concept leads to adaptor CAR T-cells having a reduced risk of tonic signaling.

Although these results support a favorable activation profile during short-term stimulation, longer-term or repetitive antigen-exposure models will be needed to determine whether these phenotypic characteristics are preserved under conditions that more closely resemble chronic stimulation within the TME of CC tissue.

Overall, the findings of this study demonstrate that PSCA-specific RevTM-mediated redirection enables efficient and tightly controlled activation of RevCAR T-cells against PSCA-expressing CC cells. Activated RevCAR T-cells acquire a cytotoxic effector phenotype as indicated by upregulation of CD69, Granzyme B, and Perforin. Activation of RevCAR T-cells induces potent, strictly antigen-dependent cytotoxicity, accompanied by secretion of the pro-inflammatory cytokines IFN-γ, TNF-α, GM-CSF, and IL-2. In addition to acquiring cytotoxic effector functions without signs of exhaustion, activated RevCAR T-cells can develop a memory phenotype. Together, our phenotypic analysis supports our original speculation that genetic modification of T-cells with RevCAR genes leads to a low level of tonic signaling. In summary, our results provide the first proof of concept for the application of the RevCAR platform in CC and support its further evaluation in more complex preclinical models incorporating heterogeneous antigen expression and the immunosuppressive tumor microenvironment.

## 4. Materials and Methods

### 4.1. Cell Lines

HEK293T and murine 3T3 cells were obtained from the American Type Culture Collection (ATCC; Manassas, VA, USA). The SiHa cell line is derived from a human cervical squamous cell carcinoma and has been widely used in research for several decades. They were originally isolated from primary uterine biopsy fragments from a 55-year-old female Japanese patient with squamous cell carcinoma. This CC cell line was genetically modified by lentiviral transduction to stably express either firefly luciferase alone or both firefly luciferase and PSCA, as previously described [131]. The resulting cells were termed SiHa-Luc^+^ or SiHa-PSCA^+^-Luc^+^ cells. SiHa, SiHa-Luc^+^, and SiHa-PSCA^+^-Luc^+^ cells, Ab-producing 3T3 cell lines, and HEK293T cells were cultured in complete Dulbecco’s Modified Eagle Medium (DMEM) [131] and were incubated at 37 °C in a humidified atmosphere containing 5% CO_2_. Mycoplasma contamination was routinely excluded by PCR-based testing.

### 4.2. Design, Expression, and Purification of PSCA RevTMs

The PSCA RevTMs were previously designed as either scFv-based [55] or IgG4-based RevTMs [55]. TM constructs generally contain a murine Igκ signal peptide (SP) at the N-terminus to enable secretion into the culture supernatant. This was followed by a PSCA-specific scFv, an scFv recognizing the La epitope E5B9, and C-terminal purification tags (Figure 1b,c).

The humanized scFv sequences specific for PSCA and E5B9 were derived from previously published mAbs MB1 and 5B9, respectively [91]. To generate an extended half-life PSCA-IgG4 RevTM, the PSCA scFv was fused to the hinge and Fc domain of human IgG4 and then combined with the VH and VL domains of the La-specific mAb 5B9 [55].

Previously established 3T3 production cell lines were used to stably express the RevTMs [55]. Cell culture supernatants were collected, and PSCA RevTMs were purified by affinity chromatography using Ni-NTA for the PSCA-scFv RevTM and Strep-Tactin for the PSCA-IgG4 RevTM. Protein purity and integrity were assessed by SDS–PAGE followed by staining with Quick Coomassie^®^ Stain (Serva, Heidelberg, Germany) or by immunoblot analysis, as previously described [91].

### 4.3. T-Cell Isolation and Generation of RevCAR T-Cells

Buffy coats from healthy human donors were obtained from the German Red Cross (Dresden, Germany) following written informed consent. Peripheral blood mononuclear cells (PBMCs) were subsequently isolated by density gradient centrifugation using Pancoll separating solution (1.077 g/mL; PanBiotech, Aidenbach, Germany), and primary human T-cells were enriched by negative selection using the Pan T Cell Isolation Kit (Miltenyi Biotec, Bergisch Gladbach, Germany) according to the manufacturer’s instructions.

For RevCAR T-cell generation, purified T-cells were activated using T Cell TransAct™ (Miltenyi Biotec) and transduced with lentiviral vectors encoding the RevCAR-E5B9 construct, as previously described [55]. During activation, transduction, and expansion, T-cells were cultured in TexMACS™ medium (Miltenyi Biotec) supplemented with recombinant human IL-2, IL-7, and IL-15 (Miltenyi Biotec). Lentiviral transduction was performed by spin infection, and transduced cells were expanded for several days under cytokine support. Transduction efficiency was assessed by the reporter gene expression (EGFP) and by surface staining with anti-La mAb (5B9). For functional assays, RevCAR T-cells were transferred to complete Roswell Park Memorial Institute (RPMI) 1640 medium and cultured without cytokines for 24 h before experiments.

The use of human T-cells was approved by the local ethics committee of the Medical Faculty Carl Gustav Carus, TUD Dresden University of Technology, Germany (approval number EK138042014), and conducted in accordance with the Declaration of Helsinki and relevant ethical regulations.

### 4.4. Flow Cytometry-Based Binding Assays

The binding of PSCA RevTMs to tumor cells and RevCAR T-cells was evaluated by flow cytometry. For this, 1 × 10^5^ cells of either the parental SiHa cell line or the genetically modified SiHa cell lines (SiHa-Luc^+^ or SiHa-PSCA^+^-Luc^+^) were seeded in a 96-well plate and incubated with the anti-PSCA mAb (MB1) [91] as a positive control or with increasing concentrations of PSCA RevTMs diluted in staining buffer (PBS/2% FBS) for 1 h. Following incubation, cells were washed, and PSCA RevTM binding was detected by a 30 min incubation with APC-conjugated anti-His mAb (Miltenyi Biotec), whereas anti-PSCA mAb binding was detected using Alexa Fluor 647 goat anti-mouse IgG (Biolegend, San Diego, CA, USA). A mouse IgG1 isotype control was used as a negative control for MB1 staining. Expression of the E5B9-RevCAR receptor on transduced T-cells was assessed using the anti-La mAb 5B9 [66], followed by Alexa Fluor 647 goat anti-mouse IgG. A corresponding mouse IgG2a isotype control was included as a negative control. All incubation steps were carried out at 4 °C, and propidium iodide was used as a viability marker. Data was acquired on a MACSQuant 10 Analyzer (Miltenyi Biotec) and analyzed using MACSQuantify or FlowJo v10 software. Binding experiments performed for K_D_ determination were conducted in technical triplicates and independently repeated at least three times.

### 4.5. Luciferase-Based Cytotoxicity Assay

The cytotoxic potential of RevCAR T-cells was analyzed using a luciferase-based assay, as previously described [132]. Luciferase-expressing target cells were seeded in 96-well plates and co-cultured with RevCAR T-cells in the presence or absence of PSCA RevTMs. The condition without RevTM served as a negative control to assess baseline target cell killing in the absence of RevTM-dependent RevCAR activation, as the RevTM is required to bridge RevCAR T-cells to PSCA-expressing target cells. To further validate the target specificity of the RevCAR system, in some assays we used the previously described anti-PD-L1 RevTM [132] as a non-specific TM, as PD-L1 expression was not detected under the assay conditions. Luminescence was recorded after 8 h, 16 h, or 24 h of incubation.

In standard experiments, 25 × 10^3^ RevCAR T-cells were co-cultured with 5 × 10^3^ target cells per well, corresponding to an E:T ratio of 5:1. A PSCA RevTM concentration of 50 nM was used unless otherwise indicated. For dose–response analyses, increasing concentrations of PSCA RevTMs were applied, and EC_50_ values were calculated by nonlinear regression analysis using GraphPad Prism version 10. Experiments assessing different E:T ratios were conducted by varying the number of RevCAR T-cells, while maintaining a constant number of target cells. Luminescence measurements were performed using a NanoQuant Infinite M200 Pro plate reader (Tecan, Crailsheim, Germany). All conditions were performed in technical triplicates, and experiments were independently repeated using RevCAR T-cells derived from three different healthy donors (n = 3).

### 4.6. Cytokine Release Assay

RevCAR T-cells were co-cultured with SiHa-PSCA^+^-Luc^+^ cells for 16 h at an E:T ratio of 5:1 in the presence or absence of 50 nM PSCA RevTM. Supernatants were harvested from the corresponding cytotoxicity assays after co-culture and analyzed for IFN-γ, IL-2, GM-CSF, and TNF using OptEIA Human ELISA kits (BD Biosciences, Heidelberg, Germany) according to the manufacturer’s protocol.

Cytokine concentrations were determined using standard curves ranging from 7.8 to 500 pg/mL, with sample dilutions adjusted to be able to determine concentration values within this range.

### 4.7. Phenotypic Analysis of RevCAR T-Cells

Following 16 h of co-culture with SiHa-PSCA^+^-Luc^+^ target cells in the presence or absence of the corresponding PSCA RevTMs, RevCAR T-cells were harvested, washed with staining buffer (PBS/2% FBS), and stained with fluorochrome-conjugated antibodies for 20 min at 4 °C. Propidium iodide was added immediately before acquisition to exclude non-viable cells. Data were acquired on a MACSQuant^®^ Analyzer 10 (Miltenyi Biotec) and analyzed using MACSQuantify^®^ or FlowJo v10 software.

For memory phenotype analysis, cells were stained with antibodies against CD4, CD8, CD45RO, CD62L, and CD95. Memory subsets were defined according to the expression of CD45RO, CD62L, and CD95 as naïve T-cells (TN, CD45RO^−^CD62L^+^CD95^−^), stem cell memory T-cells (TSCM, CD45RO^−^CD62L^+^CD95^+^), central memory T-cells (TCM, CD45RO^+^CD62L^+^), effector memory T-cells (TEM, CD45RO^+^CD62L^−^), and terminal effector memory T-cells re-expressing CD45RA (TEFF, CD45RO^−^CD62L^−^).

To evaluate antigen-induced activation, cells were stained with antibodies against CD8 and CD69. Intracellular expression of Granzyme B and Perforin was assessed after fixation and permeabilization using the Inside Stain Kit (Miltenyi Biotec) according to the manufacturer’s instructions, followed by staining with fluorochrome-conjugated antibodies against Granzyme B and Perforin.

For the analysis of exhaustion-associated markers, cells were stained with antibodies against CD8, PD-1 (CD279), TIM-3 (CD366), and LAG-3 (CD223). In addition to the individual analysis of each marker, combinatorial expression patterns were evaluated to determine the proportion of RevCAR T-cells expressing zero, one, two, or three exhaustion-associated markers.

Single-stained and fluorescence-minus-one (FMO) controls were included for compensation and gating. RevCAR T-cells were identified as viable EGFP^+^ lymphocytes before subsequent analysis of CD8 expression, activation, intracellular cytotoxic molecules, memory phenotype, and exhaustion-associated markers.

### 4.8. Statistical Analysis

Statistical analyses were performed using GraphPad Prism version 10. For cytotoxicity and cytokine release assays, statistical significance was assessed using two-tailed paired Student’s *t*-tests to compare each condition to the corresponding control without PSCA RevTM. Data are presented as mean ± standard deviation (SD) from three independent biological replicates using RevCAR T-cells derived from different healthy donors (n = 3). All experiments were performed in technical triplicates unless otherwise indicated. Differences were considered statistically significant at * *p* < 0.05, ** *p* < 0.01, and *** *p* < 0.001.

## 5. Conclusions

Collectively, these findings show that the RevCAR platform can be successfully applied for retargeting of RevCAR T cells to PSCA-expressing CC cells, supporting the feasibility of switchable CAR T-cell strategies for the treatment of this tumor entity. The precise control of T-cell activation via external RevTMs, coupled with antigen-dependent functional responses, underscores the potential of this approach to overcome critical limitations of conventional CAR T-cell therapy and to improve their clinical applicability.

## Data Availability

All data supporting the findings of this study are included in the article and Supplementary Material and are available from the corresponding author upon reasonable request.

## Supplementary Materials

The following supporting information can be downloaded at: https://www.mdpi.com/article/10.3390/ijms27219704/s1.

## Abbreviations

The following abbreviations are used in this manuscript:

AICD	Activation-induced cell death
CAR	Chimeric antigen receptor
CC	Cervical cancer
CEA	Carcinoembryonic antigen
EC_50_	Half-maximal effective concentrations
ELISA	Enzyme-linked immunosorbent assay
EpCAM	Epithelial cell adhesion molecule
E:T ratio	Effector-to-target cell ratio
Fc	Fragment crystallizable
EGFP	Enhanced green fluorescent protein
EGFR	Epidermal growth factor receptor
IFN-γ	Interferon gamma
IgG4	Immunoglobulin G4
IL	Interleukin
K_D_	Equilibrium dissociation constant
Luc	Luciferase
mAb	Monoclonal antibody
PBS	Phosphate buffer saline
PC	Prostate cancer
PLAP	Placental alkaline phosphatase
PSCA	Prostate stem cell antigen
PSMA	Prostate membrane antigen
RevTM	Reverse target module
scFv	Single-chain fragment variable
SD	Standard deviation
SDS	Sodium dodecyl sulfate
SP	Signal peptide
TAA	Tumor-associated antigen
TM	Target module
TNF	Tumor necrosis factor
VH	Variable domain of the heavy chain
VL	Variable domain of the light chain
