# Supplementary material for: Immunotargeting of Cervical Cancer Cells with the Adaptor CAR T-Cell Platform RevCAR
Source: Int J Mol Sci. 2026 Oct 29;27(21):9704. doi: 10.3390/ijms27219704 (PMC13762911; doi:10.3390/ijms27219704)
Supplement: Supplementary file 1 [file ijms-27-09704-s001.zip › ijms-4559782-supplementary.pdf]

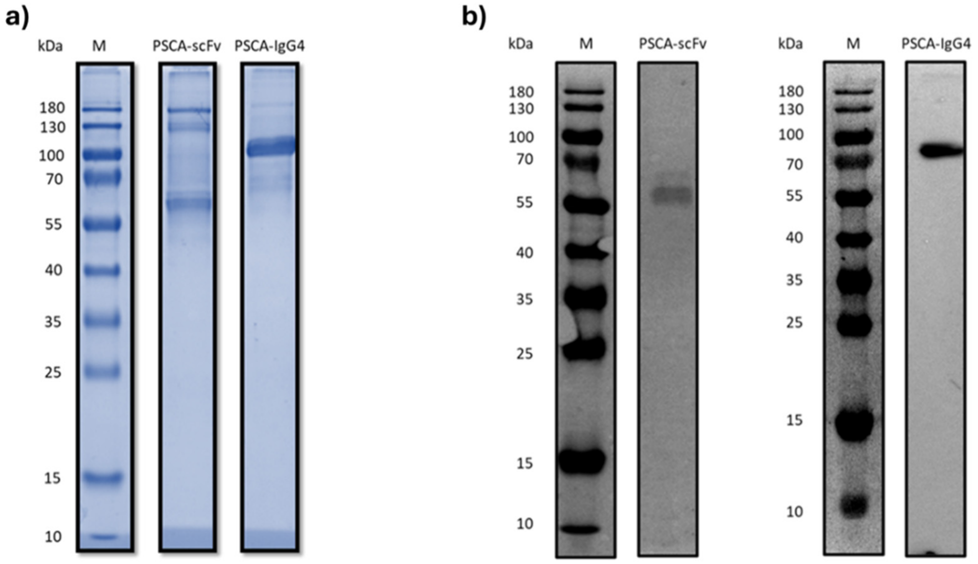

Figure S1. Biochemical characterization of PSCA RevTMs. (a) SDS–PAGE analysis of purified PSCA RevTMs under reducing conditions. (b) Immunoblot analysis of PSCA RevTMs using an anti-His antibody.

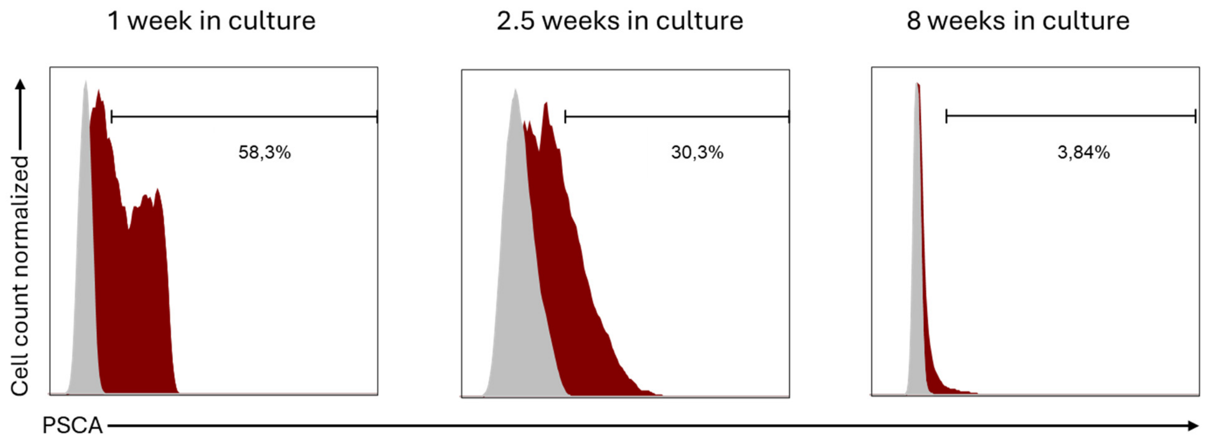

Figure S2. Decrease in endogenous PSCA expression in parental SiHa cells modified to express the luciferase gene (SiHa-Luc+ cells) during in vitro culture. PSCA surface expression was assessed by flow cytometry after 1 week, 2.5 weeks, and 8 weeks in culture using the anti-PSCA monoclonal antibody MB1. Antibody binding was detected using PE-conjugated goat anti-mouse IgG antibodies. The percentage of PSCA-positive cells decreased from 58% after 1 week to 30% after 2.5 weeks and 3% after 8 weeks in culture.

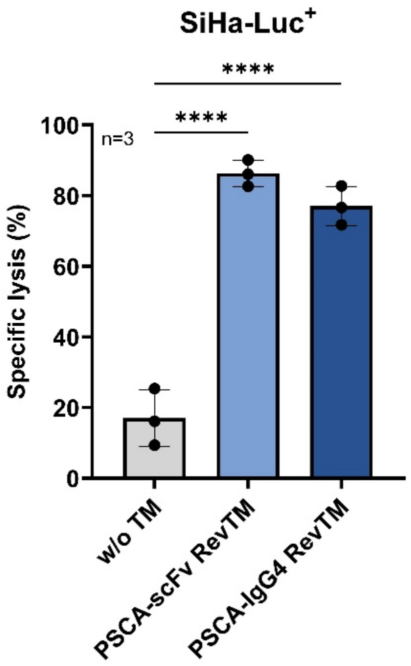

Figure S3. RevCAR-mediated cytotoxicity against parental SiHa-Luc⁺ cells expressing endogenous PSCA. Parental SiHa-Luc⁺ cells cultured for 1 week, when 58% of the population expressed PSCA, were co-cultured with RevCAR T-cells in the presence or absence of the PSCA-specific RevTM. Target cell killing was quantified using a luminescence-based cytotoxicity assay after 16 h of co-culture. Data are presented as mean ± SD from three independent experiments (n = 3).

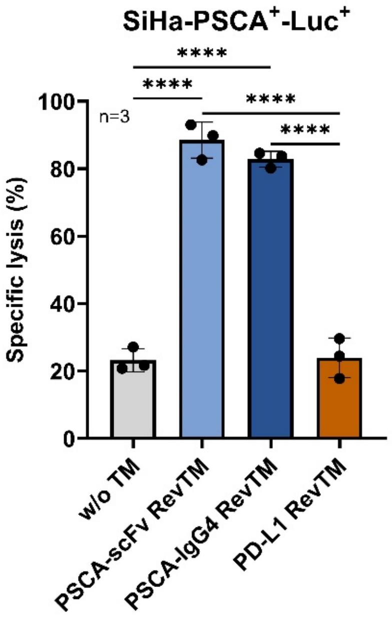

Figure S4. Assessment of antigen specificity of RevCAR-mediated cytotoxicity using a PD-L1-specific RevTM. SiHa-PSCA⁺-Luc⁺ target cells were co-cultured with RevCAR T-cells for 16 h in the presence of a PD-L1-specific RevTM. The condition without RevTM served as a negative control. PD-L1 was not detectably expressed by the target cells under the experimental conditions. Target cell lysis was quantified using a luminescence-based cytotoxicity assay. Lysis in the presence of the PD-L1-specific RevTM was comparable to that observed in the absence of RevTM. Data are presented as mean ± SD (n = 3).
